# Granular cell tumour of the pectoral muscle mimicking breast cancer

**DOI:** 10.1186/1757-1626-1-142

**Published:** 2008-09-06

**Authors:** Anish Patel, Valentina Lefemine, Syed Mansoor Yousuf, Walid Abou-Samra

**Affiliations:** 1 Speciality Registrar in Surgery, All-Wales Higher Surgical Training Programme, Glan Clwyd Hospital, Rhyl, LL18 5UJ, UK; 2 Consultant General and Breast Surgeon, Department of General Surgery, Glan Clwyd Hospital, Rhyl, LL18 5UJ, UK

## Abstract

We describe the case of a 55 year old female who presented with a mass in her right breast. Mammography confirmed a 2 × 2 cm lump, suspicious of malignancy. The lesion was widely resected. Histological examination revealed this to be a benign granular cell tumour.

Granular cell tumour is a rare tumour that very occasionally presents within the breast. It is possibly of Schwann cell origin. Clinical features and subsequent investigations may be suggestive of breast malignancy. Tumour cells showing positive immunostaining for S-100 and PAS is in keeping with the diagnosis. Wide local resection is the gold standard treatment.

## Introduction

Granular cell tumour (GCT) was first described in 1926 by Abrikosoff as a rare myogenic lesion affecting the tongue. Further immunohistochemical tests have subsequently shown this lesion to have probably a perineural or Schwann cell origin. Abrikosoff in 1931 described a similar lesion in the female breast [1]. GCT of the breast is relatively uncommon and very easily misdiagnosed for primary breast cancer. We present a case of GCT of the pectoral muscle mimicking breast cancer in a female patient, highlighting the diagnostic challenge and the treatment options in managing patients with GCT.

## Case presentation

**Figure 1:**
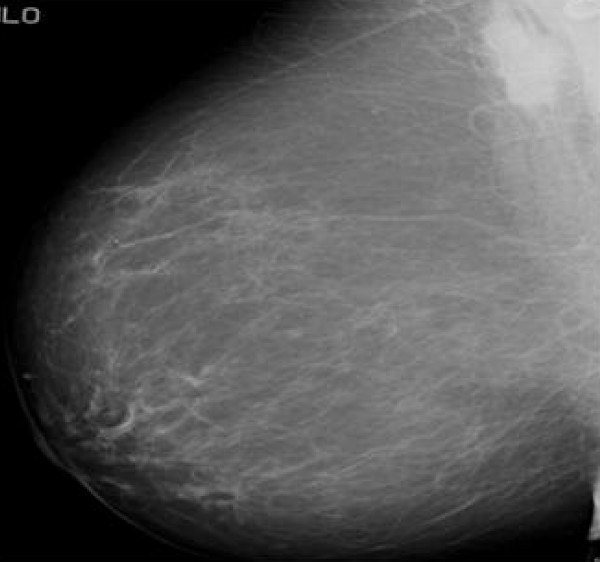
**Mammographic appearance of granular cell tumour**. Mammogram shows a dense mass deep in the upper outer quadrant of the right breast adjacent to the pectoralis major muscle.

**Figure 2:**
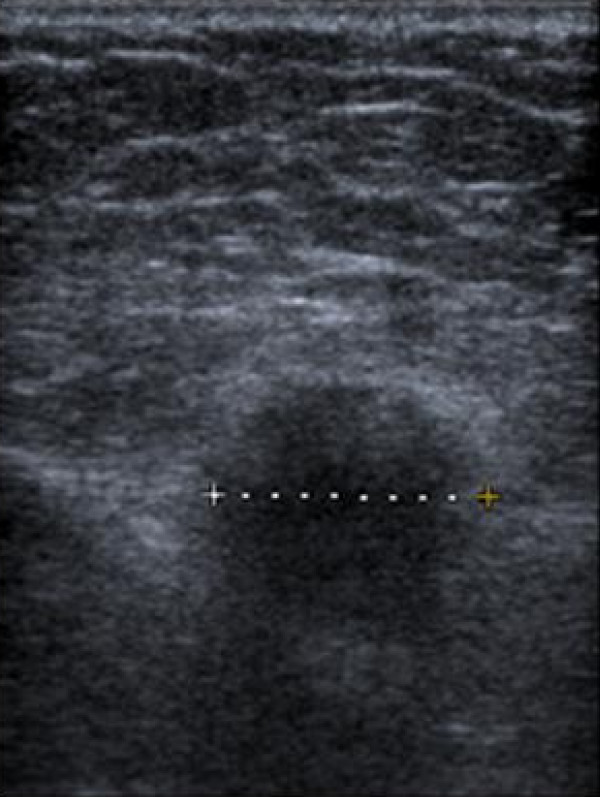
**Sonographic appearance of granular cell tumour**. Ultrasound shows an ill defined hypoechoic mass with echogenic haloing. It is difficult to differentiate this mass from a primary breast lesion, based on the sonographic findings.

A 55 year old female presented with a 2 month history of a painless lump in her upper outer quadrant of her right breast. She was on hormone replacement therapy for the previous 4 years. There was no family history of breast cancer and past medical history was unremarkable. Examination revealed a hard non mobile lump in the axillary tail of her right breast, which measured 2 × 2 cm and was highly suspicious of primary breast malignancy. Mammography and ultrasound imaging showed a suspicious lesion in the upper outer quadrant of the right breast which appeared to be attached to the underlying pectoralis major muscle [Figure 1 and 2]. As part of routine triple assessment core biopsies of the lump were taken under ultrasound guidance. Histological examination of the biopsies suggested a granular cell tumour (benign tumour of neural type differentiation/origin). Wide local excision of the tumour was performed. Macroscopically the tumour appeared to be originating from the lateral border of the pectoralis major muscle and not from the breast and was excised with an ellipse of normal muscle tissue.

Microscopically the specimen consisted of a central area containing cells with finely granular eosinophillic cytoplasm containing central bland nuclei. Immuno-histochemistry staining showed tumour cells were positive for S 100 and weakly cytopositive for PAS, in keeping with a diagnosis of granular cell tumour. The tumour was completely excised. The patient remains well 6 months after surgery.

## Discussion

GCT is a rare usually benign tumour which is most frequently encountered in the tongue, but can occur in a variety of visceral and cutaneous sites. Although there are a limited number of reported cases in the English literature the frequency of GCT is estimated to be 1 per 1000 cases of breast cancer [2]. It is more common in middle age, pre-menopausal, black women. Breast GCT in male patients is extremely rare. The preponderance of these tumours in women, especially premenopausal, has led to the hypothesis that hormones are implicated in the pathogenesis of these tumours in the breast, however, up to date, no oestrogen or progesterone receptors have been found on the tumour cells in any of the cases so far reported in the literature. GCT of the breast arises from interlobular breast stroma and affects predominantly the upper inner quadrants of the breast, in the territory of distribution of the cutaneous sensory branches of the supraclavicular nerve. GCT of the breast are usually benign but malignant cases have been described including 2 cases of GCT and infiltrating breast cancer within the same breast [3,4].

**Figure 3:**
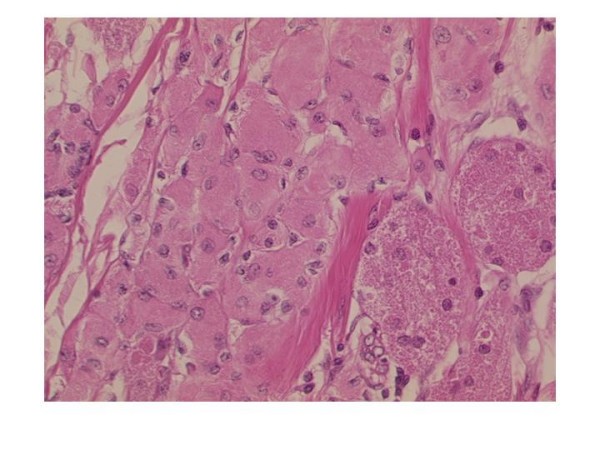
**Photomicrograph of core biopsy specimen**. Typical microscopical features of a granular cell tumour, showing nests of cells with abundant pink granular cytoplasm and small, hyperchromatic nuclei which are centrally located.

The histogenesis of GCT remains uncertain, the hypothesis of a neural or neuroectodermal origin is supported by the presence of the S-100 protein, typically expressed by these neoplastic cells, and by the similar ultrastructural features of the tumour cells and the Schwann cells. GCT are macroscopically, solid, firm tumours with a yellowish-white cross sectional surface. Microscopically they are usually characterised by clusters or sheets of polygonal cells with distinct borders and abundant granular eosinophillic cytoplasm. The nuclei are small, central and typically hyperchromatic [Figure 3]. Histological features suggestive of malignancy include tumour size > 5 cm, presence of necrosis, cellular and nuclear pleomorphism, increased mitotic activity, high nuclear to cytoplasmic ratio and large nucleoli [5].

GCT can closely resemble primary breast malignancies both clinically, due to their fibrous consistency, and radiologically. They present as hard lump which grow in an infiltrative manner and can involve the skin, causing skin dimpling and tethering, and the underlying muscle causing fixity of the lump to the deeper structures. Misdiagnosis could potentially lead to a far more aggressive treatment then necessary, hence it is important to differentiate GCT from other primary breast tumours. The final diagnosis is generally always achieved through histological examination.

Mammographic and sonographic appearance of GCT could be misleading. On mammograms GCT could present as round, well circumscribed masses, but also as indistinct densities or speculated masses which resemble primary breast carcinoma. Microcalcifications can sometimes be seen but are usually absent. The ultrasound appearances are also variable and include solid masses with indistinct margins or more benign-appearing, well circumscribed masses. Yang et al [6] has described the sonographic features of a series of 7 GCT of the breast and has interestingly shown that five of these lesions had an echogenic halo or were partially hyperechoic, this may be a result of the infiltrative growth pattern of GCT. In our case, sonographically the lesion was an ill defined hypoechoic mass with mild acoustic shadowing.

MRI scanning has proved to have additional diagnostic value in the detection of such lesions in the breast. It helps delineating the extent of the disease, the presence of aggressive features and is also valuable for concomitant screening of the controlateral breast. Kohashi et al [7] described a homogenously enhancing mass on T1 weighted imaging, the same mass showed high signal intensity rim on T2 weighted sequence. High T2 signal has been shown to be a sign of benign disease [8]. Features suggestive of malignancy include rim enhancement, speculated margins and irregular ill-defined shape of the breast lesion. Interestingly microcalcifications have been absent in every case of GCT reviewed so far and their presence should point towards a malignancy other then GCT [9].

For GCT which have been proven to be benign at core biopsy close observation is an acceptable treatment option although wide local excision is regarded as gold standard in the treatment of benign GCT. Local recurrence is associated with incomplete excision hence a complete clearance of the tumours with histologically clear margins is paramount. Axillary sampling or sentinel lymph node biopsy is not indicated [2] in the management of benign GCT as nodal invasion is extremely rare. Malignant GCT should be treated like other malignant breast tumours, however treatment has a poor overall outcome [10].

Differentiation between benign and malignant breast GCT is almost impossible on the clinical basis, and remains challenging even after accurate histological study of the specimen. High index of suspicion is therefore paramount, especially in presence of a breast mass with associated axillary lymphadenopathy, or if the breast lesion is larger then 4 cm in diameter on radiological imaging. On MRI evidence of infiltration of adjacent tissues and rim enhancement are also regarded as suspicious features.

## Conclusion

In conclusion, as GCT can easily be misdiagnosed as primary breast carcinoma clinically and radiologically, histological analysis of these lesions is needed to achieve a diagnosis. This case highlights the benefits of triple assessment in the management of beast lumps Surgeons should always be aware that GCT of the breast can resemble primary breast tumour in order to avoid performing unnecessary radical surgery in this group of patients.

## Competing interests

The authors declare that they have no competing interests.

## Authors' contributions

WAS and SMY performed the surgery. AP and VL were major contributors in the writing of the whole manuscript. All authors read and approved the final manuscript.

## Consent

Written informed consent was obtained from the patient for publication of this case report and accompanying images. A copy of the written consent is available for review by the Editor-in-Chief of this journal.
